# Geographical and practical challenges in the implementation of digital health passports for cross-border COVID-19 pandemic management: a narrative review and framework for solutions

**DOI:** 10.1186/s12992-023-00998-7

**Published:** 2023-12-08

**Authors:** Gideon Towett, R. Sterling Snead, Knarik Grigoryan, Julia Marczika

**Affiliations:** The Self Research Institute, Broken Arrow, USA

**Keywords:** Digital health passport, COVID-19, Geographical disparities, Practical challenges, Vaccines, Data security, Global standardization, Technological infrastructure

## Abstract

The rapid global spread of infectious diseases, epitomized by the recent COVID-19 pandemic, has highlighted the critical need for effective cross-border pandemic management strategies. Digital health passports (DHPs), which securely store and facilitate the sharing of critical health information, including vaccination records and test results, have emerged as a promising solution to enable safe travel and access to essential services and economic activities during pandemics. However, the implementation of DHPs faces several significant challenges, both related to geographical disparities and practical considerations, necessitating a comprehensive approach for successful global adoption. In this narrative review article, we identify and elaborate on the critical geographical and practical barriers that hinder global adoption and the effective utilization of DHPs. Geographical barriers are complex, encompassing disparities in vaccine access, regulatory inconsistencies, differences across countries in data security and users' privacy policies, challenges related to interoperability and standardization, and inadequacies in technological infrastructure and limited access to digital technologies. Practical challenges include the possibility of vaccine contraindications and breakthrough infections, uncertainties surrounding natural immunity, and limitations of standard tests in assessing infection risk. To address geographical disparities and enhance the functionality and interoperability of DHPs, we propose a framework that emphasizes international collaboration to achieve equitable access to vaccines and testing resources. Furthermore, we recommend international cooperation to establish unified vaccine regulatory frameworks, adopting globally accepted standards for data privacy and protection, implementing interoperability protocols, and taking steps to bridge the digital divide. Addressing practical challenges requires a meticulous approach to assessing individual risk and augmenting DHP implementation with rigorous health screenings and personal infection prevention measures. Collectively, these initiatives contribute to the development of robust and inclusive cross-border pandemic management strategies, ultimately promoting a safer and more interconnected global community in the face of current and future pandemics.

## Summary box

**Table:** 

• **Impact of COVID-19**: The COVID-19 pandemic and the subsequent lockdowns have had negative effects on individuals and the global economy. With the advent of vaccines, digital health passports (DHPs) have emerged as tools for restoring normalcy while safeguarding public health. Nonetheless, several geographical and practical challenges need to be addressed for their effective global implementation
• **Vaccine access disparities and regulatory variability**: It is essential to expand manufacturing capacity to address vaccine production bottlenecks, ensure equitable distribution, promote vaccine uptake by addressing hesitancy, and establish harmonized vaccine regulatory frameworks through collaboration with international stakeholders, including governments, organizations, and the scientific community
• **Variable data security and users' privacy policies**: It is imperative to adopt globally recognized privacy and data protection principles and consider the integration of blockchain technology within DHP systems to address variations in data security and privacy policies among countries
• **Global standardization and interoperability**: Achieving global standardization and interoperability necessitates the implementation of a common data format and global standards for data exchange through open-source technologies and standards. Digital diplomacy efforts can play a pivotal role in ensuring global acceptance of such standards
• **Technological access**: Offline alternatives should be explored for offline populations. However, a comprehensive and sustainable solution to the digital divide must encompass infrastructure development, internet accessibility, digital literacy programs, and equitable digital solutions for diverse populations
• **Practical challenges:** The inherent limitations of vaccination, natural immunity, and standard diagnostic tests as sole determinants of infection risk underscore the need for a comprehensive approach to pandemic management. This approach should involve a personalized assessment of an individual's risk factors for contracting and transmitting infections, complementing DHPs with rigorous health screenings, and emphasizing adherence to personal infection prevention measures

## Background

In recent decades, the frequency and severity of pandemics affecting millions of people globally have increased significantly [1]. Although there have been recent significant advances in the fields of science and medicine, the potential for infectious diseases to spread is increasing, as is the risk of outbreaks evolving into epidemics or pandemics. There are several factors contributing to this trend, including increased globalization and connectivity, which allow agents of disease to spread quickly from one part of the world to another, sometimes in a matter of hours [2]. The COVID-19 pandemic is a striking example of how outbreaks can affect millions of people worldwide. The COVID-19 pandemic not only put health systems to the test but also had a negative impact on the global community’s social and economic situation. The transport and tourism sectors bore the brunt of the virus’s impact as a result of national governments’ enactment of strict containment measures to halt its spread [3]. These measures included travel restrictions, bans on social events, house confinement, and quarantines [4]. To mitigate the effects of these control measures, people had to be safely reintegrated into the affected social and economic activities. This entailed collecting information about a person’s health status, such as recent negative COVID-19 test results [5], and later, as vaccines became available, vaccination requirements, which gave rise to COVID-19 digital health passports (DHPs; vaccine passports or certificates) [6].

This approach is based on the assumption that not everyone needs to be quarantined to control the spread of the disease. By allowing individuals with low risk of infection to move freely, the burden on the healthcare system can be reduced, and the economy can be kept running. Despite the 2005 International Health Regulations (IHR) emphasizing the need to safeguard international traffic and trade from disruption, the COVID-19 pandemic’s unprecedented worldwide effects highlighted the necessity for revising the IHR to better align with the challenges encountered during this global health crisis [7]. Consequently, COVID-19 vaccine passports were implemented worldwide in a variety of ways. For instance, Israel was among the first countries to implement mechanisms for a domestic vaccine passport policy, which included downloading a COVID-19 "green pass" onto smartphones and issuing hard copies of the cards after the booster dose. These passes enabled holders to access restaurants and other shared public spaces [8]. The European Union (EU) developed the EU Digital COVID Certificate (EU DCC) comprising recovery, vaccination, and test certificates, which allowed vaccinated EU citizens to travel within the EU [9]. International organizations also implemented initiatives; for example, the International Air Transport Association (IATA) unveiled the IATA Travel Pass, which was mainly used by airlines to verify COVID-19 test results and digital vaccine certificates [10]. Private companies were not left behind; for instance, IBM developed the IBM Digital Health Pass, which used blockchain technology to validate health credentials in a decentralized way, ensuring that only those individuals who met specific health requirements were granted access to the premises [11].

The success of vaccination has resulted in a significant decrease in daily reported cases, with many countries easing their once-stringent regulations. However, the threat of infection still exists, with the possible emergence of more infectious variants [12]. Epidemiologists also caution that climate change, habitat destruction, and increased human-animal contact significantly heighten the risk of future pandemics by increasing the potential for zoonotic spillover [13]. Coronaviruses and influenza viruses pose a high risk due to their rapid evolution, strong contagiousness via respiratory droplets, and zoonotic transmission. These viruses, with their natural reservoirs in both domestic and wild animals, have caused spillover events in the past where the virus jumps from animals to humans. Such events may continue to occur in the future. Their genetic diversity and adaptability make them potential sources of new zoonotic infections, as the viruses can evolve and adapt to new hosts [14]. Initiatives such as the Global Virome Project, which focuses on identifying potential viral threats [15], and the Coalition for Epidemic Preparedness Innovation, which secures funding to create vaccine pipelines for addressing future pandemics [16], offer promising prospects for significantly reducing the vaccine development timeline. This suggests that DHPs are poised to assume a substantial role in future pandemics as well. Thus, navigating the post-COVID-19 era requires an effective system that is responsive to emerging health threats and adaptable to unique situations in different parts of the world. The COVID-19 pandemic has led to disparities and diverse strategies among countries in their response, which are often shaped by socioeconomic factors and evolving scientific understanding [17], posing substantial challenges to the effective operation of DHPs. 

In this narrative review, we describe the geographical and practical challenges in the global implementation of DHPs for pandemic management. We also present a framework for potential solutions and provide insights into the various tools and strategies that stakeholders, including governments, international organizations, and technology providers, can employ to overcome the geographical complexities and practical challenges associated with the global implementation of DHPs. This study contributes to the development of flexible and adaptable DHP solutions capable of effectively addressing unique global situations and responding to current and emerging health threats worldwide. 

### Review criteria

We conducted a narrative review of the published literature, websites, and other online resources from January to May 2023, with the aim of collecting data available within the last four years. Our sources included PubMed, Medline, Google, Scopus, Google Scholar, and various website pages, including those from the WHO. Within these sources, we conducted English language article searches using different combinations of keywords related to (1) COVID-19 health passports, (2) pandemic management, (3) resource-rich and limited settings, (4) technology and data exchange, and (5) legal and regulatory frameworks. These keywords were aligned with our research scope and included search terms such as “COVID-19 passports,” “COVID-19 digital health passport,” “health certification,” “vaccination passports,” “vaccine verification,” “COVID-19 vaccination campaigns,” “COVID-19 testing requirements,” “privacy and security,” “health information exchange,” “challenges,” “barriers,” “facilitators,” “acceptance,” and “discrimination.” We used Boolean operators (AND and OR) to refine search results and identify relevant literature on the subject. Additionally, we employed snowballing techniques to identify relevant articles by examining references in the articles resulting from our initial search. 

The literature was screened for relevance, rigor, and substantive contributions to the overarching narrative. Subsequently, we organized the literature into thematic groups based on common trends, challenges, proposed solutions, and critical focus areas for scaling DHPs. In total, we identified and included 98 articles for this study, which are presented as follows. 

### Divergent vaccine landscape: access disparities and regulatory variability

#### Unequal access to vaccines

In the current landscape, the availability of COVID-19 vaccines has significantly improved, largely mitigating the supply constraints encountered earlier in the pandemic. However, in the context of a pandemic like COVID-19, characterized by rapid global transmission, the possibility remains that demand for vaccines could potentially outpace the available supply. The rapid mutation and evolution of the virus also introduce an additional layer of complexity, as emerging variants may necessitate modified or booster vaccines, further complicating the vaccine supply-demand equation [18]. The dynamics of vaccine distribution in the face of a rapidly spreading pandemic are inherently complex. One must consider the intricate interplay between the speed of vaccine development, production capacity, affordability, and access, which disproportionately favor developed countries [19]. In addition, the interplay of factors such as population density, healthcare infrastructure, and vaccination hesitancy adds another layer of challenges to this issue [20]. These challenges are exacerbated in areas experiencing conflict and violence, as these situations have severe repercussions on medical infrastructure and supply chains, leading to heightened vulnerability compared to regions not experiencing such conflicts or wars [21]. Moreover, marginalized communities, including those experiencing economic hardship and racial/ethnic minority groups, have consistently demonstrated lower levels of trust in vaccination programs and exhibited higher levels of skepticism [22]. The allocation of vaccines among various demographic groups, while considering high-risk populations and essential workers, also introduces further complexity to the vaccine distribution framework [23]. Consequently, vaccine passports have the potential to be discriminatory, manifesting in several ways that favor more developed countries, privileged societies, and individuals over their less affluent counterparts and marginalized communities. 

A practical solution to the vaccine production bottleneck would undoubtedly necessitate the exchange of intellectual property or technical know-how to expand manufacturing capacity. This could involve licensing agreements between pharmaceutical companies, technology transfers, or the establishment of joint ventures. By sharing knowledge and expertise, manufacturers in different countries could increase their production capabilities and contribute to a more equitable distribution of vaccines [24]. Once an adequate supply of vaccines is secured, governments need to have systems that ensure fairness and equity in deploying vaccines. In achieving fair and equitable deployment of vaccines, appropriate allocation should be determined, and vaccine uptake should be encouraged. Through collaboration with community volunteers, mobile vaccination teams successfully reached older residents in isolated villages and remote communities, resulting in a favorable outcome marked by a significant increase in participation in African countries [21]. Promoting equity within and between priority groups is also essential to mitigate the pervasive effects of ethnic and socioeconomic disparities that exist within various systems. This necessitates prioritizing historically marginalized groups, undocumented immigrants, and the homeless in vaccine allocation and outreach efforts [25].

Collaborative initiatives, such as the COVID-19 Vaccines Global Access (COVAX) facility, could play a pivotal role in addressing global vaccine inequities by facilitating the equitable distribution of vaccines to low- and middle-income countries (LMICs). COVAX is an initiative funded by developed countries, international organizations, and philanthropic entities, all with the shared goal of ensuring that vaccines reach the most vulnerable populations [26]. Moreover, LMICs should consider increased investment in strengthening and enhancing their health and cold chain facilities, possibly with support from the international community. This effort should include exploring the use of solar-powered refrigeration systems in regions with unreliable or interrupted electricity supply [27]. These facilities are vital for the effective distribution of vaccines and for monitoring vaccination campaigns, particularly in low-resource settings [28, 29]. In the context of a widespread pandemic such as COVID-19, community facilities such as churches, mosques, and other public venues, along with temporary sites like open fields equipped with tents, can serve as vaccination centers. This approach of utilizing community facilities has historically been employed to address the limitations of medical infrastructure in many African nations when combating various public health challenges, such as the Ebola outbreaks [30]. Using local community facilities as vaccination centers can also be effective in reaching individuals who may have concerns about potential surveillance by authorities [31].

Vaccine hesitancy is a multifaceted challenge that encompasses a complex interplay of factors, including individuals’ perceptions, cultural beliefs, misinformation, mistrust in health care systems, historical context, and social influences [32]. It is not a one-size-fits-all issue; rather, it varies across different communities and populations [33]. Addressing vaccine hesitancy requires investments in public health campaigns that are culturally sensitive and accessible. These campaigns should deliver accurate information in local languages, possibly through trusted community and religious leaders and accessible media platforms. These efforts should also focus on dispelling myths and misconceptions through open and empathetic communication, building trust, and reinforcing the importance of vaccination as a tool to protect not only individual health but also the collective well-being of the entire community [34]. These strategies not only promote public health but also lay the foundation for a more successful implementation of DHPs in the future. 

#### Variable vaccine authorization and approval landscape

The intricate dynamics of vaccine authorization and approval was highlighted by the COVID-19 pandemic. Regulatory agencies around the world employ varying evaluation methodologies and timelines for authorization or approval of vaccines. For instance, although some agencies accepted data from international clinical trials, others required local clinical trials for vaccine approvals [35]. This raises pertinent questions regarding the validation of vaccines for DHPs during a pandemic. If different countries have differing levels of acceptance of vaccines, the multicountry adoption of DHPs can be hindered, which creates significant challenges and confusion for individuals who seek to use DHPs for international travel and for participating in other activities. For instance, individuals could be subjected to contradictory travel and isolation restrictions. The emergence of SARS-CoV-2 variants of concern (VOC) exacerbated the complexities to the authorization and approval of COVID-19 vaccines. The variants had a significant influence on vaccine preferences around the world, with some countries voicing concerns about the efficacy of existing vaccines and favoring certain types of vaccines based on their perceived efficacy against specific variants. For example, in South Africa, the beta (B.1.351) variant led the government to favor the Pfizer-BioNtech and Johnson & Johnson vaccines over the vaccine from Oxford-AstraZeneca, despite the country participating in its trials [36]. The Gamma (P.1) variant also caused alarm in Brazil, leading to the belief that the strain was more resistant to existing vaccines [37]. In India, the Delta (B.1.617.2) variant led to the government’s emphasis on the administration of Covishield, a variant of AstraZeneca, and Covaxin, developed by Bharat Biotech [38]. These preferences altered vaccine distribution patterns and administration strategies in various regions, resulting in inequality based on access to certain vaccines, as variants influenced the types of vaccines used and certified in any location. Concerns were also voiced about how often vaccination certificates would need to be renewed and whether or how passports could be withdrawn, perhaps on short notice. 

Several factors may contribute to global variations in regulatory guidance for vaccines, including data gaps on vaccine interchangeability, the lack of a global consensus on acceptable standards for clinical trials, political considerations influencing public opinion, and geostrategic factors. Interchangeability, which refers to the ability to use different vaccines in different doses or schedules to achieve the same level of protection, poses a significant challenge to achieving global consensus and mutual recognition of these vaccines [39]. Additionally, there is a lack of global consensus regarding the parameters of acceptable human clinical trials [40]. When assessing the safety and efficacy of vaccines, different regulatory agencies may have varying standards and requirements [41]. These variations in guidelines can arise from differences in scientific perspectives, technological advancements, the capacity to conduct clinical trials, and the interpretation of available data [40]. Moreover, political considerations play a substantial role in shaping public opinion and regulatory decisions regarding vaccines. Although governments and policymakers often prioritize public health and safety, they may also face pressure to balance domestic politics, public trust, and their country’s economic interests. As a result, these political considerations may influence the speed of vaccine approval, the level of transparency in decision-making, and communication regarding the risks and benefits of specific vaccines to the public [42, 43]. Furthermore, geostrategic considerations, particularly among high-income countries, may influence regulatory decisions. A complex interplay exists between the development and distribution of vaccines and both national and international interests. Consequently, countries may seek to secure sufficient vaccine supplies for their populations while striving to gain or maintain influence in global health diplomacy [44], which can lead to varying degrees of urgency in specific vaccine authorizations, approvals, and prioritization within the regulatory processes [45].

Therefore, to tackle the variability in vaccine authorization processes amid these challenges, a multifaceted approach is required. This approach involves building scientific consensus through international collaborations, diplomatic efforts, and leveraging technology. While recent initiatives have focused on aligning technical prerequisites for registering pharmaceutical and medical device products through the International Council for Harmonization, resulting in the creation of valuable globally adopted guidelines, there has been a lack of extension of these efforts to the regulatory review procedures. The challenges encountered during crises such as the COVID-19 pandemic and other recent outbreaks, including severe acute respiratory syndrome, Middle East respiratory syndrome, Ebola, and Zika, have underscored the difficulties in coordinating cross-national regulatory responses [40].

Consequently, to achieve a more unified approach to vaccine authorization, scientific collaborations that include sharing clinical trial data, harmonizing research protocols, and promoting open scientific communication regarding vaccine authorization and approval processes among the relevant stakeholders can help build a stronger evidence base for vaccine efficacy and safety. In past epidemics, such as Ebola, a collaborative approach implemented through forums such as the African Vaccine Regulatory Forum greatly facilitated the progress and efficiency of regulatory processes in crucial areas related to Ebola vaccine development [46]. International organizations, such as the WHO, can be effective in coordinating such collaborative efforts among countries for convergence and mutual recognition. This would go a long way toward streamlining the acceptance and validation of vaccines authorized by different regulatory bodies by reducing the need for repeated mandatory vaccine assessments, which is crucial for saving time and resources during pandemics. Moreover, to guarantee the safety and efficacy of vaccines already approved for use, it is essential to put into place a robust postauthorization surveillance system to ensure real-time data gathering on adverse events and vaccine performance. This is also important for vaccine updates when variants emerge that may evade vaccine-induced antibodies, thus helping to improve the public’s confidence in vaccines [47].

In addition to building scientific consensus, science diplomacy is crucial for addressing political considerations and geostrategic factors influencing vaccine authorization decisions. Specifically, to overcome these barriers and foster positive international relations, the application of three fundamental principles of science diplomacy, as outlined by the American Association for the Advancement of Science’s Center for Science Diplomacy Program, is essential [48]. These principles include "diplomacy for science," which involves the use of diplomatic efforts and resources to facilitate international scientific and technical cooperation; "science in diplomacy," which entails the use of scientific knowledge to inform and guide foreign policy decisions; and "science for diplomacy," which leverages international scientific and technical collaborations to strengthen diplomatic relations between nations [49]. By employing these three pillars of science diplomacy, countries can enhance their ability to collaborate, navigate complex political situations, and promote global health through a strategic fusion of science and diplomacy. 

DHPs can also be designed to accommodate different vaccine authorization landscapes, including challenges posed to vaccine approval and implementation by VOC. This includes keeping the DHPs updated by tracking information on the effectiveness of a vaccine against specific variants, providing updates on vaccine recommendations from regulatory authorities and public health organizations, and considering a mechanism to cancel and renew passports with booster shots should a variant emerge that may evade vaccine-induced antibodies. In addition, to accommodate variations in vaccine authorization requirements, adaptable verification mechanisms could be considered. This includes incorporating different types of vaccines authorized by various regulatory bodies and recognizing the specific vaccines that are effective against specific variants. The system should be flexible enough to verify and authenticate the vaccination status of individuals based on the specific vaccines they have received, taking into consideration the applicable guidelines and recommendations.

### Variable data security and users’ privacy policies in countries

Privacy concerns and data protection regulations vary across jurisdictions, and striking a balance between enabling efficient DHP systems and safeguarding individual privacy rights can be challenging. For example, some countries may have stricter regulations that prioritize individuals’ privacy rights, while others may have more flexible policies [50]. This poses significant obstacles to the multicountry implementation of DHPs, as compliance with multiple jurisdictions would be necessary [51]. Additionally, as these systems often contain sensitive personal details and health information, the potential misuse of data, especially in countries with lax data protection laws, is a significant concern. This is particularly concerning in many parts of the world where the legal and regulatory frameworks governing digital health solutions (DHSs) are either lacking, poorly enforced, or not aligned with international standards [52]. Consequently, in the absence of regulatory safeguards, malicious actors may exploit these vulnerabilities to obtain sensitive health information, potentially leading to privacy violations and identity theft. These actors could include commercial entities seeking targeted marketing opportunities and criminals aiming to exploit the data for financial gains, either through its sale or by engaging in fraudulent activities. Furthermore, the information collected in the passport could be used for purposes other than those for which it was initially intended, such as tracking an individual’s movements and activities for government surveillance or even political campaigns [22].

Health data breaches can have severe social consequences, including discrimination, exclusion, and stigmatization, all stemming from the sensitive information contained in health passports [53]. For instance, employers, insurers, or other entities may access or request this information to make employment, insurance, or other decisions, potentially disadvantaging individuals based on their health status. Additionally, these concerns can lead to significant trust issues, resulting in hesitancy to embrace DHPs [54].

While there is no one-size-fits-all solution to data security and privacy, as each country has its own laws and regulations governing these issues and there are varying cultural and societal attitudes toward privacy and data protection [55], it is crucial to define regulations and mechanisms that allow access to personal information without jeopardizing the privacy of users or enabling unauthorized disclosure and use of information for inappropriate purposes. Globally recognized privacy and data protection principles that form the cornerstone of laws and regulations, such as the EU’s General Data Protection Regulation, are critical in shaping the development of responsible data practices worldwide [56]. These principles serve as guidance for countries seeking to strike a balance between data security and privacy and public health while developing their own laws and regulations. These regulations are based on the principles of lawfulness, fairness, and transparency (providing clear and accessible information about data practices to individuals); purpose limitation (using data for specific, explicit, and legitimate purposes); data minimization (retaining and collecting only necessary data); accuracy (maintaining accurate and up-to-date information); storage limitation (keeping data only for as long as necessary and in a form that does not permit the identification of individuals); data security (protecting data from unauthorized access and breaches); accountability (complying with privacy regulations and handling data responsibly); and user consent (obtaining informed and voluntary consent from individuals) [57]. By following these data protection principles and subject rights, organizations can build trust with the users of DHPs and ensure compliance with legal regulations. The establishment of independent oversight bodies at either national or regional levels, similar to the European Data Protection Board, to conduct audits and closely monitor DHSs will help ensure adherence to data protection and privacy regulations [58].

Furthermore, implementing a decentralized DHP system based on technologies such as blockchain offers several advantages in terms of data privacy and control, enabling the secure and efficient exchange of health data across borders. This approach involves distributing data across multiple nodes, which eliminates the need for a central entity to be entrusted with data storage and management. Instead, users can control their data by storing it locally or granting access to specific authorized parties, such as healthcare providers or travel authorities, when necessary [59]. This controlled data sharing fosters trust between users and data requestors because the user retains the ability to revoke access at any time. Moreover, data can be securely shared using cryptographic techniques, ensuring privacy during transmission [60, 61]. A decentralized DHP system can also enable seamless exchange between different healthcare providers, institutions, and systems by using standardized data formats and protocols, such as Fast Healthcare Interoperability Resources (FHIR), enabling efficient data exchange and sharing. This enhances healthcare coordination and decision-making [62]. Furthermore, blockchain technology, with its inherent transparency and immutability, provides a secure and tamper-resistant environment for health data storage. Each transaction and data update is recorded on the blockchain, creating an auditable trail of information. This transparency builds trust among users and relevant stakeholders, ensuring the integrity of the data and minimizing the risk of fraud or data manipulation [63]. This is particularly important considering that the falsification of vaccination records has been a significant concern, not only for COVID-19 but also for diseases such as yellow fever [64–66]. These concerns raise questions about their impact on the validity of vaccine certificates, vaccination coverage, epidemic control, and the resurgence of previously controlled diseases [67].

### Need for global standardization and interoperability

International standardization and interoperable technologies for DHPs are essential to enable cross-border credential verification to become a globally effective tool in the management of pandemics, similar to the current travel passport system. Standardization guarantees the accurate and consistent exchange of data across different systems and platforms, while interoperability frameworks define common protocols, formats, and data models, facilitating the secure and efficient sharing of health information [68, 69]. However, due to the dynamic and rapidly evolving nature of pandemics, as exemplified by the unprecedented challenges posed by COVID-19, nations faced an immediate and pressing need to develop their own, often tailored approaches to DHPs. These responses were often influenced by political considerations, with each country navigating its own unique set of circumstances. Consequently, these initiatives often had different technical specifications, data formats, and verification processes. This lack of uniformity led to fragmentation and incompatibility between different systems, resulting in confusion and inconvenience, and further exacerbating inequalities in access to travel and economic opportunities [70]. Issues of accuracy, reliability, privacy, and data security were also encountered, as the security measures used by different systems may vary, increasing the risk of mishandling or misuse of personal information, potentially compromising individuals’ privacy [71]. Such discrepancies can lead to disparities in trust and confidence in DHPs, as different countries may exhibit varying levels of scrutiny or skepticism toward foreign DHP systems. Moreover, the absence of global standards hampers international efforts to effectively respond to public health emergencies. In the context of infectious pandemics, the establishment of a global standard or interoperability framework for DHPs could facilitate the seamless exchange of accurate and up-to-date health information between countries, thereby enhancing pandemic management strategies [69].

Ongoing discussions have been held and initiatives implemented at the international level to establish common standards and interoperability frameworks for DHPs. Notable examples include the WHO response to the urgent need for global standards and specifications for COVID-19 DHPs. With the aim of developing internationally recognized standards and a trust framework for DHPs while taking into account national and international challenges, the WHO convened a Working Group for a Smart Vaccination Certificate Initiative. This initiative involved international organizations such as the United Nations Children’s Fund, the International Telecommunication Union, and the European Commission [72]. The key areas of focus for the working group included common standards and governance for authentication, privacy, security, and data exchange. The working group was dissolved in June 2021, and in August 2021 and March 2022, the WHO published documents on "Digital Documentation of COVID-19 Certificates: Vaccination Status" and "Test Results," respectively [73]. In its guidance, the WHO leveraged existing free and open standards, such as the Health Level Seven FHIR implementation, which was already well-established within the digital health domain. Additionally, other organizations, including vocabulary standards groups like SNOMED, modified their nomenclature to reflect the new codes to enable semantic interoperability [74].

Inspired by the success of regional initiatives such as the EU DCC system, which embraced open-source technologies and standards and was widely adopted by all EU Member States and 51 non-EU nations and territories, in June 2023, the WHO proposed the creation of a worldwide system aimed at safeguarding global citizens against existing and potential health crises, including pandemics. This system, known as the global digital health certification network, is a voluntary framework designed for member states to enable citizens to authenticate their health records and securely use their electronic health data [73]. However, it is crucial to acknowledge that establishing common standards and interoperability frameworks around DHPs that are globally acceptable is not just a technical or health systems issue but also a matter of building trust among different countries and stakeholders and diplomatic considerations. Digital health diplomacy, a type of science diplomacy that involves the use of digital technologies to promote global health and well-being, can be useful in this regard [75]. The Madrid Declaration for Science Diplomacy provides a framework for collaboration and communication between scientists, policymakers, and other stakeholders. It recommends the creation of interactive spaces where scientists, policymakers, and other stakeholders can come together to exchange ideas and collaborate on projects. It also promotes effective communication between scientists and diplomats and understanding of each other’s perspectives. Furthermore, it advocates for a multidisciplinary approach to science diplomacy, underscoring the importance of transparency and accessibility of scientific data and findings for all stakeholders. Additionally, it stresses the need for science diplomacy initiatives to adhere to a set of shared values and principles that prioritize the well-being of all individuals [76].

### Technological infrastructure and accessibility issues

The COVID-19 outbreak highlighted the existing disparities in access to digital technology between developed and developing countries. Countries with greater access to technology were able to leverage it to contain the spread of the virus. For instance, developed countries could employ technology to trace individuals who had come into contact with the virus, notify them of their potential exposure, and provide them with accurate, real-time information regarding the pandemic and local regulations. However, many developing countries, due to a lack of technological resources, were unable to harness these technological advancements [77, 78]. Furthermore, while developed nations swiftly transitioned to remote work and online learning, numerous developing countries grappled with limited internet connectivity and insufficient digital infrastructure [79]. DHPs require a robust and dependable digital infrastructure, including secure databases, internet accessibility, and technical components like smartphones and computers. Implementing these components may prove more challenging in LMICs with limited digital infrastructure or substantial disparities in access to digital technology. Additionally, even within developed nations with widespread smartphone adoption, certain segments of the population, such as the elderly, may exhibit reluctance or an inability to use smartphones [80]. Consequently, aside from missing out on other critical resources and solutions, populations affected by the digital divide may not be able to use DHP systems to prove their vaccination or test status, placing them at heightened risk during health crises. 

The availability and accessibility of digital technology can vary widely between different countries, regions, and even within communities in the same country. Worldwide, sociodemographic factors such as age, disability, education level, gender, and income have a significant impact on an individual’s access to digital technology. Additionally, undocumented immigrants subject to increased policing and surveillance often encounter obstacles in accessing digital technology due to the requirement to provide identification documents [54, 81]. Consequently, addressing these challenges requires a multifaceted approach that encompasses providing alternatives, infrastructure improvements, and the promotion of greater access to digital technology. 

For individuals who face a complete inability to access the internet or digital devices to use DHPs, offline alternatives can be explored. These alternatives can provide individuals with tangible proof of their health status without relying on digital infrastructure. Two potential offline options that can be considered are physical cards with a printed barcode or a unique QR code for each certificate, or an electronic health information card with a microchip [82, 83]. However, implementing these offline alternatives requires careful consideration of several factors. Preventing falsifications or counterfeits is crucial. Additionally, strong security measures must be in place to protect sensitive personal information stored on the cards or within the microchips. Furthermore, options for different local languages should be provided to accommodate diverse populations. 

Incorporating SMS and USSD services enables citizens without smartphones to access DHP information and services through text messages on their basic mobile phones [84]. Additionally, exploring the development of DHPs with offline capabilities could ensure that users can access critical information and services, especially in areas with sporadic internet connectivity [85]. To accommodate individuals with limited digital literacy, DHP interfaces should prioritize simplicity and user-friendliness, featuring clear instructions and intuitive navigation [86]. A prudent approach for these initiatives is to commence with pilot programs, which facilitate the identification and resolution of challenges before scaling the program to encompass entire communities or countries. 

While offline alternatives can serve as a temporary solution, bridging the gap for individuals who are unable to access digital platforms, a comprehensive and sustainable solution to the digital divide must unequivocally encompass efforts aimed at ensuring equal access to digital technology for all populations. This can be achieved through partnerships and effective policy frameworks designed to address the unique challenges responsible for the variable digital landscape, such as costs and a lack of skills training. Policies should include measures to make digital services available to all users, including those in remote or rural areas. This can be achieved by investing in the development of digital infrastructure, such as reliable internet access and mobile phone networks, improving access to electricity, and collaborating with technology companies, both local and international, to develop cost-effective solutions tailored to the specific needs of each country [87]. Additionally, investing in digital literacy programs and communicating the benefits of digital devices to the public encourages people, especially the less tech-savvy individuals, to use them [88]. Equitable access to digital technology has far-reaching benefits beyond the context of health passports, as it can enhance the effectiveness of controlling future pandemics and enable more equitable and efficient crisis management. 

### Practical challenges in the deployment of digital health passports

The implementation of DHPs for pandemic management presents several practical challenges. For instance, while vaccination has proven highly effective in reducing the prevalence and severity of COVID-19 infections, certain individuals may be unable to receive vaccination or complete the recommended vaccine regime due to medical contraindications stemming from allergies to specific components of vaccines, such as polyethylene glycol in mRNA vaccines [89]. Additionally, even among fully vaccinated individuals, breakthrough infections can occur, particularly in high-risk groups with comorbidities or the elderly [90]. While the risk of severe illness, hospitalizations, and death is significantly reduced in such cases, the risk of transmitting the virus to others remains [91]. This highlights the limitations of relying solely on vaccination status as a criterion for DHP issuance. Similarly, natural immunity acquired through prior infection exhibits variability in duration and strength depending on individual factors and the pathogen. Studies indicate that the natural immunity to SARS-CoV-2 is linked to the severity of the illness, with those experiencing more severe symptoms exhibiting a stronger immune response [92]. However, old age and the presence of chronic conditions can weaken the immune response, increasing susceptibility to reinfection, especially in the presence of more contagious viral strains [92]. Moreover, assessing natural immunity presents challenges, as existing antibody tests may not reliably measure an individual's protection against infection [93]. These complexities make it difficult to incorporate the concept of natural immunity into DHPs. 

Furthermore, polymerase chain reaction (PCR) tests, widely considered the gold standard for COVID-19 detection by the WHO, may not always be effective in preventing the spread of the virus or its introduction into a country. Several factors contribute to this limitation. First, the stage of the disease can significantly impact the effectiveness of PCR testing. During the incubation period and in the later stages of infection after viral clearance, viral RNA levels in the body can be very low, making detection challenging using standard PCR methods. This variability in viral load across different stages of infection suggests that individuals may be infectious even if their PCR test results are negative [94]. Second, individuals can potentially contract the virus while in transit, including at airports or during flights, after being cleared at their point of origin. This risk of post-clearance infection highlights the limitations of relying solely on PCR testing at departure points to prevent the introduction of the virus into a country. Third, observer errors arising from improper sample collection, handling, or processing can lead to inaccurate test results. Such human error can result in false-negative or false-positive test results due to mix-ups, potentially allowing infected individuals to enter a country undetected or unnecessarily restricting the travel of uninfected individuals. These limitations suggest that even if PCR tests are conducted correctly, they may still yield inaccurate results. As a result, relying solely on PCR testing as a primary prevention strategy in DHPs may not be sufficient to effectively control the spread of the virus. 

Accommodating vaccine contraindications in DHPs for specific individuals requires considering exceptions to vaccination based on individual health considerations. This entails implementing a secure and reliable verification process for medical exemptions, ensuring that such exemptions are granted solely on legitimate medical grounds. This would require collaboration with medical professionals to thoroughly assess individual circumstances and ensure that exemptions are granted only when genuinely warranted. In such cases, recent PCR tests could be considered as an alternative to vaccination. For individuals who have previously contracted COVID-19 and subsequently recovered, vaccination offers a more reliable and durable form of immunity compared to immunity acquired solely through infection. Hybrid immunity, which combines the protective effects of natural immunity and vaccine-induced immunity, has been demonstrated to provide a stronger and longer-lasting defense against reinfection compared to either natural immunity or vaccination alone [95].

The occurrence of vaccine breakthrough infections and the inherent limitations of standard diagnostic tests highlight the need for a more comprehensive approach to pandemic management. This involves a thorough and individualized assessment of an individual's risk factors for contracting and transmitting infections, considering factors such as travel history, vaccination status, and potential exposure to the virus. Furthermore, complementing DHPs with other preventive measures, such as stringent health screenings, hand hygiene practices, mandatory mask-wearing, and social distancing protocols at airports, on flights, and in shared public spaces [96], should remain in place to further minimize the risk of transmission. Public awareness and education campaigns can play a crucial role in managing expectations and clarifying that DHPs, while valuable, are not foolproof safeguards. They should be viewed as one layer of defense against the virus, with continued adherence to public health guidelines and personal precautions remaining essential. 

To ensure consistent and reliable COVID-19 test results, it is imperative to adhere to established standard test procedures set forth by reputable organizations like the WHO. These protocols provide essential guidelines and best practices that help maintain the accuracy and integrity of testing processes, thus eliminating the risk of observer errors [97]. Nevertheless, implementing such procedures can pose a formidable challenge in many developing countries, where limited resources and infrastructure may hinder the establishment of standardized testing facilities, potentially affecting the quality and accessibility of testing services [98].

When recent tests are required, it is imperative to ensure equal access to fast and reliable testing for DHPs to function effectively. Due to the interconnected nature of global health, where a health threat in one region can have repercussions worldwide, support in the form of financial aid, technical assistance, and the provision of necessary equipment and supplies from developed nations and international organizations, along with collaborations with various agencies, becomes vital in assisting LMICs in building the essential infrastructure and developing capacity for testing, disease surveillance, and the distribution of testing resources to underserved communities and remote areas. 

## Conclusion

The review of geographical and practical challenges faced by DHPs reveals the intricate obstacles hindering their effective global adoption. Addressing disparities in vaccine accessibility demands concerted efforts to enhance vaccine development, distribution, and acceptance. Navigating the diverse landscape of vaccine authorization and approval landscapes requires a multifaceted approach that encompasses international collaboration to establish common guidelines and standards for vaccine authorization and approval, science diplomacy, scientific consensus building on clinical trial data and research protocols, and leveraging technology in the design of DHPs to accommodate different vaccine authorization landscapes and the emergence of variants. Balancing the crucial need for health data exchange with safeguarding individuals' privacy rights necessitates harmonized policies and universally recognized data protection principles to guarantee trust and compliance. Additionally, by exploring the potential of decentralized technologies, DHP systems could be developed to prioritize individual privacy rights while still enabling the seamless exchange of information required for efficient DHPs. Bridging the digital divide through investments in digital infrastructure, such as expanding broadband access and providing digital literacy training, alongside the provision of offline DHP alternatives, will contribute to ensuring universal access to DHPs. Due to the limitations of vaccination, natural immunity, and standard tests in solely determining infection risk, DHPs should be considered as one component of a comprehensive infection prevention strategy. Additional measures, such as rigorous health screenings, mask-wearing, social distancing, and hand hygiene, should be implemented to further minimize the risk of transmission. By implementing these strategies, stakeholders can collectively work towards overcoming the barriers to universally applicable DHP systems for effective pandemic management. Continued research, evaluation, and refinement of DHP systems are vital to ensuring their effectiveness and adaptability in the face of evolving public health challenges.

## Data Availability

Not applicable.

## Abbreviations

COVAX: COVID-19 vaccines global access
DHP: Digital health passport
DHS: Digital health solutions
EU: European Union
EU DCC: European Union Digital COVID Certificate
FHIR: Fast healthcare interoperability resources
IATA: International Air Transport Association
IHR: International Health Regulations
LMIC: Low- and middle-income countries
PCR: Polymerase chain reaction
VOC: Variants of concern
WHO: World Health Organization
